# Characteristics of trauma mortality in the Northern Territory, Australia

**DOI:** 10.1186/s40621-017-0111-1

**Published:** 2017-05-15

**Authors:** Kathleen M. McDermott, Matt B. Brearley, Steven M. Hudson, Linda Ward, David J. Read

**Affiliations:** 1 National Critical Care and Trauma Response Centre, Level 8 Royal Darwin Hospital Rocklands Drive, Tiwi, NT 0810 Australia; 2 Gisbourne Hospital, Gisbourne, New Zealand; 3 Menzies School of Health Research, Darwin, Australia; 4 Royal Darwin Hospital, Darwin, Australia

**Keywords:** Trauma, Mortality, Indigenous, Season, Remote, Injury severity score

## Abstract

**Background:**

While factors including remoteness, alcohol consumption, age and Indigenous ethnicity are well-documented associations of trauma mortality, less is known of trauma seasonality. This is particularly relevant to Australia’s Northern Territory, with its tropical regions experiencing a climate of wet (hot and humid) and dry (warm) seasons annually. The aim of this study was to therefore, examine the characteristics of trauma mortality in the Top End, Northern Territory, Australia.

**Methods:**

A retrospective review of the National Coroners Information System (NCIS) database from 1 January 2003 to 31 December 2007 analysed four-h﻿undred and sixt﻿een﻿ traumatic deaths where the trauma event and death occurred within the Top End of the Northern Territory.

**Results:**

The annual traumatic death rate for the Top End was 58.7 per 100 000, with variance between regions (accessible 38.1; remote 119.1 per 100000, respectively). Overall alcohol was involved in 56.5% of cases. The three most frequent mechanisms of death were suicide, transport related and assault, accounting for 81.5% of deaths. These respective mechanisms of death demonstrated seasonal influence, with transport related deaths 2.5 times more likely to occur in the dry than the wet season (*p* < 0.001), while assault related deaths were 3.3 times more likely to occur during the wet season (*p* = 0.005), and suicide was 1.6 times more likely to occur during the wet season (*p* = 0.022). Transport related deaths were 2.2 times more likely in remote and very remote settings than in accessible or moderately accessible regions (*p* < 0.003), whereas death by suicide was less likely to occur in remote and very remote regions than in accessible or moderately accessible areas (*p* = 0.012).

**Conclusion:**

Excessively high rates of traumatic death in the Top End of the Northern Territory were evident, with contrasting seasonal﻿ and regional profiles. Based upon the data of this investigation, existing programmes to minimise trauma in the Northern Territory ought to be evaluated for seasonal and regional specificity.

## Background

It is well established that injury mortality increases with remoteness from major cities (Danne 2003; Fatovich and Jacobs 2009; Mitchell and Chong 2010), is the second highest cause of death for Indigenous people (Australian Institute of Health and Welfare (AIHW) 2007) and that alcohol consumption is correlated to injury mortality (Demetriades et al. 2004). These factors are particularly relevant to the Northern Territory (NT), Australia, where mean alcohol consumption equates to 14 L per capita, approximately 50% higher than national average (Skov et al. 2010) Population demography in the NT is unique with a median age of 30.5 years (Australian Bureau of Statistics 2013), the highest proportion of children aged 0–15 years (24.5%) and the lowest proportion of 65 years and over (4.5%) nationally (Australian Bureau of Statistics 2006). Indigenous people represent approximately 30% of the total NT population (Australian Bureau of Statistics 2013), in contrast to less than 4% for other states and territories (Australian Bureau of Statistics 2006). Approximately three quarters of the NT population (~142000) (Australian Bureau of Statistics 2013) reside in a region termed the ‘Top End’, defined as the area north of the township of Elliott. The Top End encompasses approximately 500 000 square kilometres (Gowing et al. 2015) including the capital city of Darwin, the Katherine region to the south and Arnhem region to the east producing a very low population density (Australian Bureau of Statistics 2013). The majority of the Top End population live within the urban and rural areas, with just 23.5% of the population residing in the remote and very remote areas (Australian Bureau of Statistics 2013).

Climate is a defining feature of the Top End, with two distinct seasons experienced annually, the ‘Wet’ and ‘Dry’. The wet season (October – March) is characterised by hot temperatures, high humidity and monsoonal rainfalls, creating harsh outdoor conditions.

Conversely, the dry season (April – September) is known for warm sunny days with low humidity and cool nights, providing ideal conditions for outdoor activities and is therefore peak tourism season with a tourist population approaching half a million annually (Tourism NT: Quickstats report 2016).

The Top End is serviced by three public hospitals, Royal Darwin Hospital, a 350 bed tertiary referral hospital, Katherine District Hospital, a regional 60 bed acute care facility, and Gove District Hospital, a regional 32 bed acute care facility. The sole private hospital of the region is based in Darwin and does not accept direct major trauma admissions. The region has multiple remote area health clinics and retrieval to hospital is achieved by road or air. Of all Australian states and territories the NT has the highest annual injury mortality rate of an age adjusted 97.2 deaths per 100 000 (2004/05 report), in excess of twice the national average (Henley and Harrison 2004). While alcohol consumption and the Top Ends hot climate are well documented, less is known of traumatic death rates of this region, and whether death from traumatic injury is associated with remoteness, severity of injury and access to medical services.

Furthermore, seasonal trends are yet to be identified. Therefore, the purpose of the study was to descriptively report injuries resulting in death in the Top End of the Northern Territory and to analyse for what variables are associated with mortality.

## Methods

### Inclusion and exclusion criteria

All NT trauma related deaths were obtained from the National Coroners Information System. Injury locality within postcodes 0800–0850 and 0854 were automatically included. Injuries sustained within postcodes 0852 and 0862 were individually analysed based upon specific location as these postcodes traverse the border of the Top End and Central Australia.

Deaths from traumatic injuries sustained outside of the Top End and deaths occurring interstate from injuries sustained in the Top End were also excluded. Mechanism of injury was classified according to external cause of injury codes in ICD-10-AM.

### Data analysis

Cases that met the inclusion criteria were analysed for the following characteristics: age, gender, Indigenous status, mechanism of injury, injury date and time, alcohol involvement, level of medical care, and injury time to death. Mechanism of injury was classified as assault, burn, drowning, electrocution, suicide, transport and other. Transport related injury represented involvement of any form of transport in the death, inclusive of motor vehicle, motorbike, bicycle and pedestrian. Location of death was classified as at scene, remote health clinic, regional hospital or Royal Darwin Hospital. The location of residence, injury and death were also analysed post classification through the use of the Accessibility/Remoteness Index for Australia (ARIA) (Hugo et al. 1999; DHAC (Department of Health and Aged Care) and GISCA (NATIONAL Key Centre for Social Applications of Geographical Information Systems) 2001). Remoteness of a locality was based on the travel distance required to access a full range services. Darwin, the primary service centre of the Top End is classified as accessible (3.00) the minimal ARIA score of the Top End. The Injury Severity Score (ISS) was calculated from autopsy reports using the Abbreviated Injury Scale (AIS) 2005 revision, 2008 updates (Association for the Advancement of Automotive Medicine 2008). For inpatient deaths where autopsy was not performed, AIS was assigned by Trauma Clinical Nurse Consultants accredited in AIS and ISS coding from patient medical records, operative and radiology reports.

Alcohol was deemed to be involved if the coroner reported textual information of alcohol involvement in either the deceased or the person(s) involved, and/or blood alcohol was positive. The former was obtained by reviewing coronial reports of the events leading up to death for terms such as ‘alcohol intoxication’, ‘alcohol’, ‘intoxicated’ or ‘inebriated’.

### Population

Top End population was derived from the Australian Bureau of Statistics (ABS) Census data. The 2006 census data was provided on a postcode basis, with the population of postcodes 0800–0850 and 0854 included. The lower boundary of the Top End region dissects postcodes 0852 and 0862, therefore limiting the automated calculation of Top End population from these jurisdictions. While census data from 2011 was outside of the analysis period, the 2011 data provided individual population centre data not available from the 2006 census. The population of those localities within the Top End from postcodes 0852 and 0862 were calculated as a proportion of total postcode population. The resultant percentage was applied to 2006 census data to approximate the population of postcodes 0852 and 0862 within the Top End region.

### Statistical analysis

Stata version 13 (Stata Corporation, Texas, USA) was used for the statistical analyses. Chi squared or Fisher exact tests were used to assess categorical variables; *p* < 0.05 was considered significant. Multivariate logistic regression analyses with stepwise backward elimination were used to estimate the associations between the mechanism of death outcomes and the predictor variables of remoteness, Indigenous status, season and alcohol involvement.

## Results

The Top End population for 2006 was 141,761, with 68.2% of residents classified as living within the accessible region, compared to moderately accessible (8.3%), remote (7.6%) and very remote regions (15.9%). The Top End population was comprised of 24.1% Indigenous, 67.1% non-Indigenous with 8.8% not accounted for. Of the Indigenous population, 28.8% resided in the accessible region, with 8.9, 11.2 and 51.1% living in moderately accessible, remote and very remote settings, respectively.

A total of 416 trauma related deaths occurred in the Top End region during the 2003–2007 period, with the greatest proportion occurring in the accessible region (44.2%). Overall, the traumatic death rate was 58.7 persons per 100000 annually, with the accessible region exhibiting the lowest rate (38.1 per 100000), and remote region demonstrating the highest rate (119.1 per 100000), approximately three times greater than the accessible region (Table 1). The vast majority of deceased were NT residents (97.8%). Suicide, transport and assault mechanisms accounted for 81.5% of deaths, with alcohol involved in 56.5% of cases.

**Table 1:** Traumatic death descriptors for the Top End and subregions

	All regions	Accessible	Moderately accessible	Remote	Very remote
Population	141761	96671	11784	10751	22554
Traumatic deaths	416	184	53	65	114
Annualised traumatic death rate (per 100000)	58.7	38.1	91.6	119.1	101.1
Gender					
Male	319 (76.7)	139 (75.5)	46 (86.8)	43 (66.2)	92 (80.7)
Female	97 (23.3)	46 (25.3)	8 (15.1)	21 (32.3)	22 (19.3)
Ethnicity					
Indigenous	198 (47.6)	56 (30.4)	6 (11.3)	43 (66.2)	92 (80.7)
Non-Indigenous	217 (52.2)	128 (69.6)	48 (90.6)	21 (32.3)	22 (19.3)
Unknown	1 (0.2)	1 (0.5)	0 (0.0)	0 (0.0)	0 (0.0)
Age Group					
< 16 years	28 (6.7)	5 (2.7)	3 (5.7)	6 (9.2)	14 (12.3)
≥ 16 years	388 (93.3)	179 (97.3)	50 (94.3)	59 (90.8)	100 (87.7)
Mechanism					
Assault	44 (10.6)	20 (10.9)	4 (7.5)	9 (13.8)	11 (9.6)
Drowning	24 (5.8)	10 (5.4)	3 (5.7)	8 (12.3)	3 (2.6)
Falls	28 (6.7)	20 (10.9)	2 (3.8)	1 (1.5)	5 (4.4)
Suicide	148 (35.6)	72 (39.1)	24 (45.3)	17 (26.2)	35 (30.7)
Transport	147 (35.3)	53 (28.8)	17 (32.1)	26 (40.0)	51 (44.7)
Other	25 (6.0)	9 (4.9)	4 (7.5)	4 (6.2)	8 (7.0)
ISS					
Not identified	8 (1.9)	2 (1.1)	2 (3.8)	0 (0.0)	4 (3.5)
1–15	14 (3.4)	8 (4.3)	0 (0.0)	2 (3.1)	4 (3.5)
16–24 (serious)	15 (3.6)	10 (5.4)	1 (1.9)	2 (3.1)	2 (1.8)
25–39 (severe)	235 (56.5)	113 (61.4)	30 (56.6)	36 (55.4)	56 (49.1)
40–75 (critical)	144 (34.6)	51 (27.7)	20 (37.7)	25 (38.5)	48 (42.1)
Alcohol Involvement	235 (56.5)	103 (56.0)	23 (43.4)	42 (64.6)	67 (58.8)

Parentheses denote percentage of deaths for the given region

Alcohol was involved in 75.3% of Indigenous deaths and 39.3% of non-Indigenous deaths.

**Fig. 1 ﻿:**
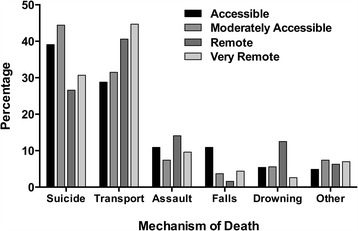
The proportion of deaths in the respective regions of the Top End stratified by mechanism of injury

Death was most frequent at the scene of injury (79.1%), compared to 17.8% at Royal Darwin Hospital, and 3.1% at regional hospitals and remote health clinics. Overall, the Indigenous (47.6%) and non-Indigenous (52.2%) proportion of deaths were similar, however Indigenous deaths accounted for 26.1% of accessible and moderately accessible, and 76.4% of remote and very remote mortality, respectively. Mechanism stratified by remoteness is shown in Fig. 1.

### Mechanism of death

#### Suicide

Of the 148 deaths due to suicide, 122 were caused by hanging (82.4%) and 14 were firearm related (9.5%). Males accounted for 87.8% of suicide related deaths, a significantly higher proportion compared to all other deaths (*p* < 0.001). The median age of deceased was 33 years, with the age categories of 16–24, 25–34 and 35–44 years accounting for 72.3% of cases. Approximately 60% of suicides occurred during the wet season. After taking remoteness, Indigenous ethnicity and alcohol involvement into account, suicide was 1.6 times more likely to occur during the wet season (*p* = 0.022) (Table 2).

**Table 2:** Bivariate and multivariate analyses of suicides

Suicide (*n* = 148)		Bivariate analyses			Multivariate analyses					
		*n*	%	*p*	Model building			Final model		
					*p*-values					
					Model 1	Model 2	Final	Odds ratio	OR 95%	
									Confidence interval	
Gender	Female	18	18.6	<0.001	<0.001	<0.001	<0.001	3.3	1.84	5.87
	Male	130	40.8							
Season	Dry	59	30.0	0.024	0.02	0.019	0.022	1.6	1.07	2.50
	Wet	89	40.6							
Remoteness	A + M	96	40.5	0.023	0.021	0.057	0.033	0.6	0.41	0.96
	R + VR	52	29.1							
Alcohol involvement	No	73	39.2	0.095	0.02	0.052	0.075	0.7	0.45	1.04
	Yes	75	31.9							
Age group	<16 years old	6	21.4	0.151	0.173	0.201	(removed)			
	≥16 years old	142	36.6							
Ethnicity	Indigenous	64	32.3	0.184	0.202	(removed)				
	Not Indigenous	84	38.7							

*A* accessible, *M* moderately accessible, *R* remote, *VR* very remote

Death by suicide was less likely to occur in remote and very remote regions than in accessible or moderately accessible areas (*p* = 0.033) (Fig. 1, Table 2). With alcohol involved in 50.7% of cases, suicide related deaths were 1.4 times less likely to have alcohol involvement than other mechanisms, although statistical significance was not achieved (*p* = 0.075). Suicide was not related to Indigenous status after accounting for season, remoteness and alcohol involvement (*p* = 0.202).

#### Transport

Of the 147 transport related fatalities, 105 were male (71.4%) and six were non NT residents (4.1%). The median age of deceased was 32 years, with the age categories of 16–24 (21.1%), 25–34 (27.2%) and 35–44 years (23.8%) respectively accounting for the vast majority of deaths. Single vehicle rollovers were identified in 43 (29.3%) cases and 40 of the deceased (27.2%) were pedestrians. Eight motorcyclists and two cyclists died during the analysis period, while three deaths were related to aircraft and one death related to marine craft. Of the 92 applicable cases, seatbelts were worn on 23 occasions (25.0%), not worn on 48 occasions (52.2%) and not determined in 21 instances (22.8%).

Alcohol was involved in 65.1% of all transport related deaths, with alcohol involvement 2.6 times more likely than for non-transport related deaths (*p* < 0.0001). Transport related deaths were 2.4 times more likely in remote and very remote settings than in accessible or moderately accessible regions (*p* < 0.004) (Table 3). Transport related deaths demonstrated a seasonal influence, as they were 2.5 times more likely to occur in the dry than the wet season (*p* < 0.001) (Table 3).

**Table 3:** Bivariate and multivariate analyses of transport related deaths

Transport (*n* = 147)		Bivariate analyses			Multivariate analysis			
		*n*	%	*p*	*p*	Odds ratio	OR 95% Confidence interval	
Gender	Female	42	43.3	0.069	0.010	0.5	0.30	0.85
	Male	105	32.9					
Season	Dry	90	45.7	<0.001	<0.001	0.4	0.25	0.59
	Wet	57	26.0					
Remoteness	A + M	70	29.5	0.004	0.001	2.4	1.43	4.03
	R + VR	77	43.0					
Alcohol involvement	No	52	28.2	0.107	<0.001	2.6	1.54	4.23
	Yes	95	40.4					
Ethnicity	Indigenous	72	36.4	0.758	0.005	2.2	1.27	3.96
	Not Indigenous	75	34.6					
Age group	<16 years old	15	53.6	0.042	0.034	0.4	0.17	0.93
	≥16 years old	132	34.0					

*A* accessible, *M* moderately accessible, *R* remote, *VR* very remote

#### Assault

The cohort of assault related deceased were residents of the NT with ages ranging from 14 to 58 years (median 35 years). Males represented 50.0% of the cohort, a significantly lower proportion than for other mechanisms (*p* < 0.001) (Table 4). Alcohol was involved in 79.5% of assault related deaths and 75.0% of victims were Indigenous.

**Table 4:** Bivariate and multivariate analyses of assault related deaths

Assault (*n* = 44)		Bivariate analyses			Multivariate analyses					
		*n*	%	*p*	Model building			Final model		
					*p*-values					
					Model 1	Model 2	Final	Odds ratio	OR 95%	
									Confidence interval	
Gender	Female	22	22.7	<0.001	<0.001	<0.001	<0.001	0.3	0.13	0.51
	Male	22	6.9							
Season	Dry	9	4.6	<0.001	<0.001	<0.001	<0.001	4.3	1.95	9.62
	Wet	35	16.0							
Alcohol involvement	No	9	5.0	0.002	0.121	0.074	0.059	2.3	0.97	5.27
	Yes	35	14.8							
Ethnicity	Indigenous	33	16.6	<0.001	0.005	0.006	0.011	0.4	0.16	0.79
	Not Indigenous	11	5.1							
Age group	<16 years old	1	3.6	0.340	0.328	(removed)				
	≥16 years old	43	11.1							
Remoteness	A + M	24	10.1	0.749	0.286	0.289	(removed)			
	R + VR	20	11.1							

*A* accessible, *M* moderately accessible, *R* remote, *VR* very remote

Multivariate analysis adjusting for both Indigenous status, gender and alcohol involvement revealed assault related deaths were 4.3 times more likely to occur during the wet season (*p* < 0.001), and that victims were 2.5 times more likely to be Indigenous than non Indigenous (*p* = 0.011).

After taking into account Indigenous status, gender and season, alcohol involvement trended towards association with assault related deaths (*p* = 0.059).

#### Falls

Of the fall related deaths, the majority were males (85.7%) and non-Indigenous (64.3%). The median age of 41 years (range 5–88 years) for fall related deaths was significantly higher than non-fall related deaths (*p* = 0.003). The accessible region accounted for 71.4% of fall related deaths, a significantly higher proportion compared to moderately accessible, remote and very remote regions (*p* = 0.003). There was a trend for lower alcohol involvement in fall related deaths compared to non-fall related deaths (*p* = 0.074).

#### Drowning

Males accounted for 19 of the 24 (79.2%) drowning deaths. Median age of the deceased was 36.5 years (range 3–79 years), with half the cohort classified as Indigenous and alcohol involved in 58.3% of cases. In the remote region, drowning accounted for 12.3% of all traumatic deaths, significantly higher than other regions (*p* = 0.012).

#### Other mechanisms

Included in the other mechanism of death category (6.0% of all deaths) were six electrocutions, five deaths as a direct result of animals, inclusive of four crocodile attacks, and three burns related deaths. In four cases, the mechanism of death was not determined.

#### Injury severity and location of death

Injury severity score (ISS) categories 1–15 and 16–24 accounted for 7.1% of all traumatic deaths. Comparing deaths within the combined 1–24 ISS between accessible/moderately accessible and remote/very remote setting revealed no differences for locality (*p* = 0.603).

**Fig. 2:**
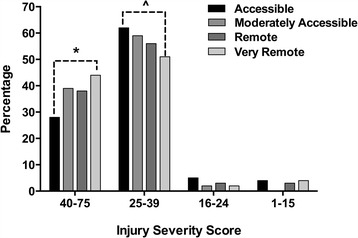
Relative percentage of injuries classified by severity score for the respective regions. * indicates very remote significantly higher than accessible (*p*<0.007). ^ indicates accessible trended higher than remote (*p*=0.062)

Suicide accounted for the largest proportion (56.6%) of the ISS category 25–39, while 77.8% of deaths in the ISS 40–75 category were transport related. In very remote settings, 44% of deaths had an ISS of 40 or greater (Fig. 2), a significantly higher proportion compared to the accessible region (*p* = 0.007) due to the high proportion of transport related fatalities. After taking into account mechanism of death, the ISS was not different across regions (*p* = 0.711).

There was a significant association between remoteness (*p* < 0.001), suicide (*p* < 0.001), other mechanisms of death category (*p* = 0.001), injury severity score (*p* < 0.001), Indigenous status (*p* = 0.002), assault (*p* = 0.003) and death at scene of injury in a multivariate analysis. Death at scene of injury was 3.9 times more likely in remote and very remote settings than in accessible or moderately accessible regions (*p* < 0.001). Alcohol was 1.9 times more likely to be involved with death at the scene (*p* = 0.064).

## Discussion

Several studies have examined injury mortality in the Northern Territory. However, little focus has been placed on quantifying injury mortality by regional profile and season. Revealing a death rate of 58.7 per 100 000 (2003–2007), multiple trends of interest are highlighted in this study.

Particularly novel to this study is the identification of seasonal associations for the three leading mechanisms of death, suicide, transport and assault. Given that suicide in the NT has been approximately double that of the national average (Australian Bureau of Statistics 2010; Pridmore and Fujiyama 2011), the relatively high frequency of suicide was anticipated. The predominance of suicide within the accessible and moderately accessible regions supports Top End data from 1991-1998 (Parker and Ben-Tovim 2002) but is in contrast to National data identifying greater suicide frequency in rural areas (Henley and Harrison 2004; Australian Bureau of Statistics 2010). That suicide was not related to Indigenous status (Parker and Ben-Tovim 2002) and alcohol was involved in approximately half of suicides (Measey et al. 2006) also corroborates earlier research findings. Yet the aetiology of suicide in the Top End is complicated by a previous unidentified seasonal association, with increased likelihood to occur during the wet season. The cause of this apparent seasonal influence is beyond the scope of this report, however, a similar seasonal trend was identified for assault related deaths. The harsh climate of the Top Ends build up and wet season contributes to heat stress (Brearley et al. 2015; Brearley et al. 2016), with residents using the colloquial terms of ‘mango madness’ and ‘troppo season’ interchangeably with ‘build up’ to describe the October to December period. The term ‘build up’ reflects the accumulation of environmental moisture during the day in combination with hot temperatures. In contrast, ‘mango madness’ and ‘troppo season’ are references to behaviour anecdotally synonymous with the oppressively hot and humid weather. While more data is required to confirm that the Top Ends environmental conditions contribute to altered behaviour and ultimately increased assault and suicide, ambient temperature has been linked with suicide (Deisenhammer et al. 2003; Kim et al. 2011; Lee et al. 2006; Volpe et al. 2008) and to a lesser extent, assault related deaths (Sisti et al. 2012) in other jurisdictions.

Whilst the second most frequent mechanism, transport related fatalities accounted for the majority of traumatic deaths in the remote and very remote regions of the Top End, and were twice as likely in the remote compared with the accessible regions. Single vehicle rollovers accounted for a relatively large proportion of transport related deaths (29.3%), with contributing factors proposed to be straight but unsealed roads, dry season, poorly maintained vehicles, excessive driving speeds and being of Indigenous ethnicity (Treacy et al. 2002). The current investigation confirms that single vehicle rollovers were more frequent in the dry season (~63%), and that unsealed roads are a likely factor given that that majority occurred in remote/very remote settings (~63%). Whilst over-represented, the majority of deceased due to single vehicle rollovers were not of Indigenous ethnicity (47%). Although the proportion of single vehicle rollovers varies by season or geography, it is not possible to compute true rates as the number of vehicles or kilometres driven is unknown. It is evident that low seatbelt usage is an area for continued road safety focus given that seat belts were not utilised in at least ~52% of applicable transport related deaths. The majority of victims were Indigenous (~56%), with Clapman et al (Clapman et al. 2008) attributing low seatbelt usage to education programmes not reaching the Indigenous community for various societal reasons.

Indigenous road safety education programmes require consideration of social determinants and disadvantage in addition to the immediate causes of injury (Clapman et al. 2008; Thompson et al. 2009). However, it is also clear that road safety needs to be addressed to the whole community, and address the role of other factors, inclusive of alcohol given its association with lack of seatbelt usage (~52% of cases). Pedestrian fatalities representing ~27% of transport related deaths was approximately twice the national average of 13% for all Australian road users (Bureau of Infrastructure et al. 2015). Factors contributing to this discrepancy may include alcohol consumption and general carelessness for road safety (Thompson et al. 2009). That involvement of alcohol in transport fatalities was twice as likely than other mechanisms of death is alarming, and may be attributed to the high consumption of alcohol in the NT at 14 L per capita (Skov et al. 2010). The majority of transport deaths occurred during the dry season. As this is the peak tourist season, it is a common held belief that the excess traffic due to the tourist population contributes to transport related deaths. Such a viewpoint is supported by interstate and overseas residents representing 16% of the whole of NT road fatalities (Northern Territory Road Injury Statistical 2013). However this investigation reveals for that the Top End this was not the case, with non NT residents accounting for just 4% of transport related deaths. For the period of this study, the NT road toll (26.2 per 100 000) was almost twice that of the leading OECD nation and three times the national average (Northern Territory Road Safety Task Report). It is likely that factors contributing to the high prevalence of transport deaths, and in particular those in the remote and very remote regions are multifactorial. A lack of speed limits, long travelling distances, infrequent traffic and a sparseness of retrieval assets and health infrastructure have been highlighted as threats to the survivability of the remote transport incident victim (Read 2015).

Furthermore, remote living Indigenous persons face further barriers, such as limited public transport options, language barriers to licencing and driver training, cultural requirements to travel at short notice en masse, a predominance of unsealed roads and limited access to vehicular maintenance (Helps et al. 2008).

During the initial four years of the study period the NT had open speed limits on rural stretches of highways, permitting drivers to freely select the speed they travelled (Northern Territory Road Safety Task Report). A road safety taskforce was established in 2006 to identify methods of reducing mortality and serious injury on NT roads (Northern Territory Road Safety Task Report). Among the taskforces recommendations was establishing speed limits on open stretches of NT highways. Indeed, the NT government introduced speed limits of 130 km/h on the open stretches of major highways and speed limits of 110 km/h on roads outside of urban areas on January 1st 2007 (Burns 2007). The impact of this initiative on transport related trauma measured by a comprehensive interrupted time series analysis is yet to be reported in the scientific literature. Assault was the sole mechanism of death where males were not over represented, with equal representation of the sexes. While the gendered nature of assault deaths is a well supported phenomenon (Mouzous and Houliaris) the equal distribution reported by this study is contrary to the findings that Australian homicide victims are overwhelmingly male (Mouzous and Houliaris; Mouzous and Seragarae; Mouzous; Davies and Mouzous; Virueda and Payne). It is beyond the scope of this paper to describe the classification of the assaults. However, the National Homicide Monitoring Programme (NHMP) (Mouzous and Houliaris; Mouzous and Seragarae; Mouzous; Davies and Mouzous; Virueda and Payne) Homicide in Australia annual report states that during 2003–2007 domestic violence was responsible for the majority of homicides in NT. Following an inquest into the death of an Indigenous female victim in the setting of repeated domestic violence, the NT Coroner made a recommendation that regulators consider mandatory reporting by health professionals of domestic violence (NTMC 083, 2006) (Inquest into the death of Jodie Palipauminni). In 2009 an amendment was passed to the *Domestic and Family Violence Act, 2007* making it mandatory for adults to report to police if harm has occurred or the reasonable belief that it will occur to persons in a domestic relationship. The data from this investigation provides a benchmark to compare data from post introduction of legislation. Assault victims were 4.6 times more likely to be Indigenous with Indigenous people representing 75% of this cohort. Overall, Indigenous people accounted for almost half (47.6%) of the traumatic deaths reported by this study. With a Top End Indigenous population of 24.1% over the study period, the Indigenous deaths are a clear over representation. As expected, Indigenous status was strongly associated with death in the remote and very remote given an Indigenous population of 67.2 and 80.7% in those respective regions. Indigenous health and injury is complex, as is developing Indigenous health promotion and injury prevention programs (Ivers et al. 2008). The health differences between Indigenous and non Indigenous people are vast. The life expectancy for Indigenous people is ten years less than non Indigenous and the rates of comorbidities are considerably higher (Australian Indigenous 2017). This report does not describe Indigenous health inequity and social determinants of health in the deserved detail, as a combination of social, economic, environmental and cumulative traumatic historical experiences underpin the inequity (Whelan and Wright 2013). These factors in combination with the large Indigenous population that is unique to the NT, and the typical remoteness of residence illustrate that there is much to be done towards improving Indigenous mortality rates. Most importantly and challenging is ensuring the cultural sensitivity of health care providers when addressing these needs (Plani and Carson 2008).

Remote and very remote settings of the Top End are diverse with small populations dispersed across large geographical areas. The leading mechanism of injury in the collective remote regions was transport. Typically, transport related deaths were associated with high ISS, however when taking into account the mechanism of injury, ISS was not different across the regions. The remoteness of injury is associated with death at the scene and the reason for this was likely the long discovery time of the injured (Fatovich and Jacobs 2009), poorer access to medical facilities (Kmet and Macarthur 2006) and that most remote region accidents are transport related, and therefore the victims are more severely injured. It is well recognised that specialised medical care for the trauma patient should be delivered within the ‘Golden Hour’ (McNicholl 1994), however, it is evident that for those injured in the remote regions such an opportunity is generally unlikely.

Alcohol is a well-described risk factor for trauma mortality (Demetriades et al. 2004; Thompson et al. 2009) and for admission to trauma centres (Macleod and Hungerford 2011). Furthermore, Indigenous Australians are at higher risk of alcohol related harm, with Vos et al (Vos et al. 2003) estimating that it accounted for 6.2% of the burden of disease in Indigenous persons. Alcohol misuse was identified as the leading risk factor for injury in Indigenous persons, and reducing harmful alcohol usage along with other risk factors, was identified as having the greatest potential to reduce the Indigenous health gap (Vos et al. 2003). With alcohol involved in ~57% of traumatic deaths, and ~75% of Indigenous traumatic deaths, alcohol remains a high risk factor for trauma related deaths and a key priority for education in the community.

### Limitations/future research directions

This study was a population based audit that quantified all pre hospital and in hospital deaths from trauma in an entire regional trauma system. Strengths of this study include the almost universal post mortem availability, ISS calculation, and the fact that Royal Darwin Hospital is the sole referral hospital for major trauma in the region. It is possible that some deaths may have been missed, if the deceased was injured in the NT, then was transferred interstate and subsequently died. The age of the data implies that any application to current times should be cautious, but this study will serve as a valuable baseline to assess the impact of subsequent trauma prevention initiatives locally. Throughout the study period there were a number of legislative changes and implementation at both the Territory and Federal level of government and recommendations made by the NT coroner in his coronial findings. Future research should include the analysis of the effect of these changes. Notably the introduction of speed limits on open stretches of highway on January 1, 2007 (Read 2015) changes in perimeter pool fencing laws (Swimming Pool Safety 2014). The 2006 recommendation by the coroner of mandatory reporting of domestic violence when introduced in 2009 (Family and Domestic Violence Act 2007s 124) (Domestic Violence Act 2007) was the first of its kind in Australia (Northern Territory Department of Children and Families 2012) and has potential to effect offender recidivism.

The introduction of the federal government Northern Territory Emergency Response Act 2007 (Northern Territory National Emergency Response Act (No 129.^2007^) 2007) in August 2007 followed the introduction of a range of broad ranging measures to Indigenous communities in the NT on June 21, 2007 ‘In response to the national emergency confronting the welfare of Aboriginal children in the Northern Territory’ (Brough 2007).

There were multiple measures introduced, including ‘introducing widespread alcohol restrictions on NT Aboriginal land and increasing policing levels in prescribed communities’ (Brough 2007). The effects of this legislation on Indigenous communities is beyond the scope of this report, however at a minimum it is obvious that this legislation would affect the social landscape of remote communities (Australian Human Rights Commission) for example internal migration. The effects of this legislation on trauma mortality in the Top End communities are yet to be measured.

## Conclusion

Contrasting regional profiles for traumatic death in the Top End of the NT are detailed by this report. The remote region demonstrated the highest annual traumatic death rate of 119.1 per 100000 persons, approximately three times greater than the accessible region. Transport related deaths were more likely to occur in remote and very remote settings, whereas suicide was more prominent in accessible and moderately accessible regions. Alcohol was involved in 56.5% of deaths. Indigenous status was strongly associated with transport and assault mechanisms. A distinct seasonal influence was apparent for suicide and assault deaths that warrants further investigation. Based upon the data of this investigation, a regional and seasonal approach to injury prevention programmes is warranted.

## Abbreviations

ARIA: Accessibility/remoteness index for Australia
ISS: Injury severity score
NT: Northern Territory
RDH: Royal Darwin Hospital

## References

[CR1] Association for the Advancement of Automotive Medicine (2008). The abbreviated injury scale, 2005 – update 2008.

[CR2] Australian Bureau of Statistics (2006). Population distribution, Aboriginal and Torres Strait Islander Australians, 2006.

[CR3] Australian Bureau of Statistics. Population by Age and Sex, Australia. 2006. Canberra: ABS 2006. (ABS Cat no.3235.0). http://www.abs.gov.au/ausstats/abs@.nsf/Products/3235.0~2006~Main+Features~Main+Features?OpenDocument. Accessed 4 Jan 2015.

[CR4] Australian Bureau of Statistics. Suicides, Australia, 2010. Canberra: ABS 2010. (ABS Cat no. 3309.0). http://abs.gov.au/ausstats/abs@.nsf/Products/3309.0~2010~Chapter~Suicide+in+Australia?OpenDocument. Accessed 4 Jan 2015.

[CR5] Australian Bureau of Statistics. 2011 Census of Population and Housing. Customised Data Report. 2013. Canberra: ABS; 2013.

[CR6] Australian Human Rights Commission. Social Justice Report 2007 - Chapter 3: The Northern Territory 'Emergency Response' intervention. https://www.humanrights.gov.au/publications/social-justice-report-2007-chapter-3-northern-territory-emergency-response-intervention. Accessed Jan 4 2015.

[CR7] Australian Indigenous HealthInfoNet (2017). Overview of Aboriginal and Torres Strait Islander health status 2016. http://www.healthinfonet.ecu.edu.au/health-facts/overviews. Accessed 1 Feb 2017.

[CR8] Australian Institute of Health and Welfare (AIHW). Leading causes of mortality: AIHW; 2007. http://www.aihw.gov.au/WorkArea/DownloadAsset.aspx?id=644245866. Accessed 15 Jun 2015.

[CR9] Brearley M, Harrington P, Lee D, Taylor R. Working in hot conditions-a study of electrical utility workers in the northern territory of Australia. J Occup Environ Hyg. 2015;12(3):156–62. 10.1080/15459624.2014.957831 25265189

[CR10] Brearley M, Norton I, Rush D, Hutton M, Smith S, Ward L, Fuentes H (2016). Influence of chronic heat acclimatization on occupational thermal strain in tropical field conditions. J Occup Environ Med.

[CR11] Brough M. Minister for Families, Community and Indigenous Affairs. Australian Government. National Emergency response to protect children in the NT [Media release] 21 June 2007. http://www.formerministers.dss.gov.au/3581/emergency_21june07/. Accessed 10 May 2015.

[CR12] Bureau of Infrastructure, Transport and Regional Economics (BITRE) Pedestrians and Road Safety, Information Sheet 70, BITRE, Canberra. 2015. http://bitre.gov.au/publications/2015/files/is_070.pdf. Accessed 2 Jan 2016.

[CR13] Burns C. Northern Territory Acting Minister for Transport. Northern Territory Government-New Road Changes Will Protect Families [Media Release]. 1 Jan 2007. http://www.territorystories.nt.gov.au/bitstream/10070/83539/1/Burns-010107-New_road_rules.pdf. Accessed 7 Jun 2015.

[CR14] Clapman K, Senserrick T, Ivers R, Lyford M, Stevenson M (2008). Understanding the extent and impact of Indigenous road trauma. Injury.

[CR15] Danne P (2003). Trauma management in Australia and the tyranny of distance. World J Surg.

[CR16] Davies M, Mouzous J. Homicide in Australia 2005–2006, National Homicide Monitoring Program Annual Report. Canberra: Australian Institute of Criminology. www.aic.gov.au/media_library/publications/rpp/77/rpp077.pdf. Accessed 12 Jun 2015.

[CR17] Deisenhammer EA, Kemmler G, Parson P (2003). Association of meteorological factors with suicide. Acta Psychiatr Scand.

[CR18] Demetriades D, Gkiokas G, Velmahos G, Brown C, Murray J, Noguchi T (2004). Alcohol and illicit drugs in traumatic deaths. Prevalence and association with type and severity of injuries. J Am Cll Surg.

[CR19] DHAC (Department of Health and Aged Care) & GISCA (National Key Centre for Social Applications of Geographical Information Systems). Measuring remoteness: accessibility/remoteness index of Australia (ARIA). Occasional papers: new series no.14. Canberra: DHAC; 2001.

[CR20] Family and Domestic Violence Act 2007. Northern Territory of Australia. http://www.austlii.edu.au/au/legis/nt/num_act/dafva200734o2007300/. Accessed 2 May 2015.

[CR21] Fatovich D, Jacobs I (2009). The relationship between remoteness and trauma deaths in Western Australia. J Trauma.

[CR22] Gowing C, McDermott K, Ward L, Martin B (2015). Ten years of trauma in the ‘top end’ of the Northern Territory, Australia: a retrospective analysis. Int Emerg Nurs.

[CR23] Helps YLM, Moller J, Kowanko I, Harrison JE, O’Donnell K, de Crispigny C. Aboriginal People Travelling Well: Issues of safety, transport and health, Adelaide: Department of Infrastructure, transport, regional development and local government. 2008. (Road Safety Report 2008–01). https://infrastructure.gov.au/roads/safety/publications/2008/RSRG_1.aspx. Accessed 6 Jun 2015.

[CR24] Henley G, Harrison J. Injury Deaths, Australia 2004–05. Injury research and statistics series no 51. 2009. Canberra: AIHW (Cat no.INJCAT 12). http://www.aihw.gov.au/WorkArea/DownloadAsset.aspx?id=6442458853. Accessed 6 Jun 2015.

[CR25] Hugo GJ, Bamford E, Dunne L (1999). Accessibility remoteness index of Australia (ARIA). Department of Health and Aged Care occasional papers series No.6. Commonwealth department of health and aged care.

[CR26] Inquest into the death of Jodie Palipauminni [2006] NTMC 083. www.localcourt.nt.gov.au/docs/judgements/2006/20061023ntmc083.html. Accessed 11 Jul 2015.

[CR27] Ivers R, Clapman K, Senserrick T, Lyford M, Stevenson M (2008). Injury prevention in Australian Indigenous Communities. Injury.

[CR28] Kim Y, Kim H, Kim DS (2011). Association between environmental temperature and suicide mortality in Korea (2001–2005). Psychiatry Res.

[CR29] Kmet L, Macarthur C (2006). Urban–rural differences in motor vehicle crash fatality and hospitalisation rates among children and youth. Accid Anal Prev.

[CR30] Lee HC, Lin HC, Tsai SY, Li CY, Chen CC, Huang CC (2006). Suicide rates and the association of climate: a population-based study. J Affect Disord.

[CR31] Macleod JB, Hungerford DW (2011). Alcohol related injury visits: do we know the true prevalence in U.S trauma centres?. Injury.

[CR32] McNicholl BP (1994). The golden hour and pre hospital trauma care. Injury.

[CR33] Measey MA, Li SQ, Parker R, Wang Z (2006). Suicide in the Northern Terriory. 1981–2002. Med J Aust.

[CR34] Mitchell RJ, Chong S (2010). Comparison of injury related hospitalized mortality in urban and rural areas in Australia. Rural Remote Health.

[CR35] Mouzous J. Homicide in Australia 2003 *–* 2004, National Homicide Monitoring Program Annual Report. Canberra: Australian Institute of Criminology. http://www.aic.gov.au/media_library/publications/rpp/66/rpp066.pdf. Accessed 6 Jun 2015.

[CR36] Mouzous J, Houliaris T. Homicide in Australia 2004 *–* 2005, National Homicide Monitoring Program Annual Report. Canberra: Australian Institute of Criminology. www.aic.gov.au. Accessed 6 Jun 2015.

[CR37] Mouzous J, Seragarae M. Homicide in Australia 2002 *–* 2003, National Homicide Monitoring Program Annual Report. Canberra: Australian Institute of Criminology. www.aic.gov.au. Accessed 6 Jun 2015.

[CR38] Northern Territory Department of Children and Families. Evaluation of The Impact of Mandatory Reporting of Domestic and Family violence Northern Territory, 2012. https://justice.nt.gov.au/__data/assets/pdf_file/0012/171201/evaluation-impact-mandatory-reporting-domestic-family-violence.pdf. Accessed 3 Mar 2015.

[CR39] Northern Territory National Emergency Response Act (No 129.2007) 2007. Australian Government. http://www.comlaw.gov.au/Details/C2007B00157?Explanatory%20Memorandum/Text. Accessed 3 Mar 2015.

[CR40] Northern Territory Road Injury Statistical Summary 2015. https://nt.gov.au/__data/assets/pdf_file/0005/229055/nt-road-crash-statistical-summary.pdf. Accessed 11 Apr 2017.

[CR41] Northern Territory Road Safety Task Report: Safer Road Use: A Territory Imperative. Northern Territory Government. http://www.territorystories.nt.gov.au/bitstream/10070/81080/4/Martin.24.10.06.road_safety_report.recommendations.pdf. Accessed Accessed 6 Jun 2015

[CR42] Parker R, Ben-Tovim DI (2002). A study of factra affecting suicide in aboriginal and ‘other’ populations in the top end of the Northern Territory through an audit of coronial records. Aust N Z J Psychiatry.

[CR43] Plani F, Carson P (2008). The challenges of developing a trauma system for indigenous people. Injury.

[CR44] Pridmore S, Fujiyama H (2011). Suicide risk factors for NSW. Aust NZ J Psychiatry.

[CR45] Read DJ (2015). Open speeds on Northern Territory roads: not so fast. Med J Aust.

[CR46] Sisti D, Rochi MB, Maccio A, Preti A (2012). The epidemiology of homicide in Italy by season, day of week and time of day. Med Sci Law.

[CR47] Skov S, Chrikritzhs T, Li Shu Q, Pircher S, Whetton S (2010). How much is too much? Alcohol consumption and related harm in the Northern Territory. Med J Aust.

[CR48] Swimming Pool Safety Act, 2014. Northern Territory Government. http://www.austlii.edu.au/au/legis/nt/consol_act/spsa244/. Accessed 2 Jan 2015.

[CR49] Thompson N, Krom I, Ride K. Summary of road safety among Indigenous peoples. 2009. https://www.healthinfonet.ecu.edu.au/uploads/docs/road-review.pdf. Accessed 6 Jun 2015.

[CR50] Tourism NT: Quickstats report. Northern Territory Government. 2016. http://www.tourismnt.com.au/corporate/research/latest-visitor-data. Accessed 11 Apr 2017.

[CR51] Treacy PJ, Jones K, Mansfield C (2002). Flipped out of control. Med J Aust.

[CR52] Virueda M, Payne J. Homicide in Australia 2007 *–* 2008, National Homicide Monitoring Program Annual Report. Canberra: Australian Institute of Criminology. http://www.aic.gov.au/media_library/publications/mr/mr13/mr13.pdf. Accessed 6 Jun 2015.

[CR53] Volpe FM, Tavaris A, Del Porto JA (2008). Seasonality of three dimensions of mania:psychosis, aggression and suicidality. J Affect Disord.

[CR54] Vos T, Barker B, Stanley L, Lopez AD. The Burden of Disease and Injury in Aboriginal and Torres Strait Islander peoples 2003. Brisbane School of Population Health. Brisbane: The University of Queensland.

[CR55] Whelan S, Wright DJ (2013). Health services and use and lifestyle choices of indigenous and non-indigenous Australians. Soc Sci Med.

